# Numerical Investigation of Graphene and STO Based Tunable Terahertz Absorber with Switchable Bifunctionality of Broadband and Narrowband Absorption

**DOI:** 10.3390/nano11082044

**Published:** 2021-08-11

**Authors:** Yan Liu, Rui Huang, Zhengbiao Ouyang

**Affiliations:** 1 THz Technical Research Center of Shenzhen University, Shenzhen Key Laboratory of Micro-Nano Photonic Information Technology, Key Laboratory of Optoelectronic Devices and Systems of Ministry of Education and Guangdong Province, College of Physics and Optoelectronic Engineering, Shenzhen University, Shenzhen 518060, China; yanliu0923@163.com; 2 School of Materials Science and Engineering, Hanshan Normal University, Chaozhou 521041, China; rhuang@hstc.edu.cn

**Keywords:** terahertz absorber, dynamically tunable, switchable bifunctionality, excellent absorptance

## Abstract

A graphene metamaterial and strontium titanate (STO)-based terahertz absorber with tunable and switchable bifunctionality has been numerically investigated in this work. Through electrically tuning the Fermi energy level of the cross-shaped graphene, the bandwidth of the proposed absorber varies continuously from 0.12 THz to 0.38 THz with the absorptance exceeding 90%, which indicates the functionality of broadband absorption. When the Fermi energy level of the cross-shaped graphene is 0 eV, the proposed absorber exhibits the other functionality of narrowband absorption owing to the thermal control of the relative permittivity of STO, and the rate of change of the center frequency is 50% ranging from 0.56 THz to 0.84 THz. The peak intensity of the narrowband absorption approximates to nearly 100% through adjusting the Fermi energy level of the graphene strips. The calculated results indicate that it is not sensitive to the polarization for wide incidence angles. The proposed absorber can realize tunable bifunctionality of broadband absorption with a tunable bandwidth and narrowband absorption with a tunable center frequency, which provides an alternative design opinion of the tunable terahertz devices with high performance for high-density integrated systems.

## 1. Introduction

Metamaterials, artificially engineered by subwavelength electromagnetic materials, show some optical properties that differ from natural materials. The resonance frequency can be arbitrarily customized in a large frequency range in the microwave [1,2], terahertz (THz) [3,4,5], and near-infrared regions [6]. Metamaterial-based absorbers (MMAs) have attracted great interest due to their scalable properties and a wide variety of potential applications, for example solar and thermophotovoltaic energy conversion [7,8], cloaking [9,10], sensors [11], and thermal emitters [12,13]. Conventional MMAs are composed of a sandwich structure with a dielectric spacer, which acts as a divider between the subwavelength metallic patterns and a continuous metallic ground plane. Since the first perfect MMA was proposed and investigated [14], MMAs with single- [15,16], dual- [17], multi- [18,19], and broad-band [20,21,22] absorption have been proposed and investigated. However, the absorption performance of MMAs influenced by the geometric parameters of unit cells cannot be dynamically adjusted anymore once the fabrication is completed.

To enrich the functionalities, active materials are introduced into metamaterial devices, such as vanadium dioxide [23], phase-change materials [24], liquid crystals [25], liquid metals [26], and especially graphene [27,28]. Due to its extraordinary physical properties [29,30], such as high electron mobility, flexible tunability [31], relatively low loss, and tight field confinement, graphene has become a very promising material for many technologies [32,33,34]. Due to the visibility of monolayer graphene being much stronger in reflection than that in transmission [35], optical reflection microscopy is more frequently used to identify the layers, size, and position of graphene, which directly determines the quality of the graphene-based devices. Many methods, such as using a thick substrate with a sizeable oxide layer [35], imaging ellipsometry [36], spin-coating with polymethylmethacrylate (PMMA) [37], and surface plasmon resonance reflectance [38], have been used to detect and characterize the graphene on different substrates. Notably, two-dimensional (2D) polymers (2DPs), in addition to graphene, have been successfully prepared in the experiment, which shows an alternative approach for future electronics and energy-related applications [39]. Strontium titanate (STO) material is a typical functional ceramic dielectric material that shows many special physical properties [40,41], such as low dielectric loss, superior insulation, good chemical stability, and thermal control of the dielectric constant, among which the most interesting one is that its dielectric permittivity can be dynamically changed through controlling the external environment temperature. Recently, tunable devices with multiple functionalities have been investigated including the bifunctional absorbers transforming from broadband absorption to narrowband absorption. However, the absorption cannot maintain a stable intensity when the operating bandwidth or center frequency *f_c_* is adjusted in a large range of frequencies. A comparison of the tunability of the bifunctional absorber and other terahertz devices with correlative functionalities are shown in Table 1. The parameters for comparison include the bandwidth (BW), center frequency *f_c_*, and rate of change (ROC, dividing the change in operating bandwidth or frequency by the original BW or *f_c_*, respectively).

In this study, a graphene metamaterial and STO-based absorber with switchable and tunable bifunctionality is proposed in the terahertz regime. Through controlling the Fermi energy level of the cross-shaped graphene, a tunable broadband absorption of the bifunctional absorber can be obtained, and its bandwidth can realize a variation in the range of frequencies from 0.12 to 0.38 THz with excellent absorptance of over 90%. The relative impedance as well as the field analyses are investigated to reveal its absorbing mechanism. When the Fermi energy level of the cross-shaped graphene is 0 eV, a narrowband absorption with tunable center frequencies can be achieved utilizing thermal control of the relative permittivity of STO. Furthermore, the effects of the geometrical parameters and various incidence angles for different polarization on absorption performances are also discussed. The bifunctional absorber inspires the design of dynamically tunable devices with multiple functionalities in the terahertz regime.

## 2. Materials and Methods

**Figure 1:**
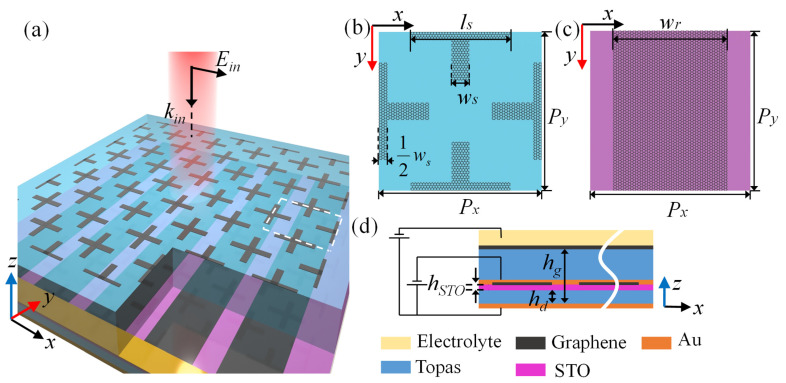
(**a**) The structural schematic and the polarization configuration of the incident wave of the designed absorber. Top view for (**b**) the upper metamaterial and (**c**) the lower metamaterial. (**d**) Side view of the bifunctional absorber with the electrolyte gate.

The structural schematic and polarization configuration of the incident wave of the bifunctional absorber is shown in Figure 1a, which is composed of a cross-shaped graphene metamaterial layer, an insulating spacer (polyethylene cyclic olefin copolymer, Topas) [48], a 1.9-μm-thick STO layer covered by strip-shaped graphene metamaterial, and a bottom gold film with conductivity of 4.56 × 10^7^ S/m used as the continuous metallic reflector. The thickness of the gold film is 0.5 μm, which is much greater than the maximum skin depth conductivity of the gold film. Topas, a transparent and stiff amorphous thermoplastic copolymer, shows superior optical properties such as high stability, excellent heat resistance, negligible absorption coefficient, and constant refractive index in the THz range [49]. The relative permittivity of Topas is 2.35 with negligible loss and dispersion in this work [50]. Thicknesses of the insulating spacer *h_g_* and *h_d_* are 40 μm and 6.4 μm, respectively. Figure 1b,c shows the upper and lower metamaterial of the bifunctional absorber, respectively. The branch length and width of the cross-shaped graphene is *l_s_* = 36 μm and *w_s_* = 6 μm, respectively, and the width of the graphene strips is *w_r_* = 42 μm. The periods of the unit cell for the bifunctional absorber are *P_x_* = *P_y_* = 60 μm. The side view is depicted in Figure 1d, where the cross-shaped graphene metamaterial is covered by a 20-μm-thick electrolyte (poly-(ethylene oxide)/LiClO_4_) layer with refractive index 1.7 [51].

The CVD-grown graphene layer is transferred onto the multilayer substrate by a transfer technique using polymethylmethacrylate (PMMA) supporting layers and is subsequently patterned by photolithography and oxygen plasma etching [52]. The graphene layer can be modeled as a surface current defined as *J* = *σ_g_E_t_* according to Ohm’s law, where *E_t_* is the tangential electric field, and *σ_g_* is the complex conductivity of graphene. The surface conductivity of graphene from Kubo formula is determined by the combination of intraband and interband contributions [53]:

(1) $$\sigma_{g}\approx \frac{je^{2}}{4\pi \hbar }ln\left[\frac{2\left|\mu_{c}\right|-\left(\omega +j/\tau \right)\hbar }{2\left|\mu_{c}\right|+\left(\omega +j/\tau \right)\hbar }\right]+j\frac{e^{2}k_{B}T}{\pi \hbar^{2}\left(\omega +j\tau^{-1}\right)}\left[\frac{\mu_{c}}{k_{B}T}+2\ln\left(\exp\left(-\frac{\mu_{c}}{k_{B}T}\right)+1\right)\right]$$

where *ω* is the incident angular frequency, *k_B_* ≈ 1.381 × 10^−23^ J/K is the Boltzmann constant, *e* ≈ 1.602 × 10^−19^ C is electron charge, *ħ* ≈ 1.055 × 10^−34^, J·s is the reduced Planck constant, and *T* is temperature in kelvin. The values of chemical potential *μ_c_* and Fermi energy level *E_f_* are equal when *k_B_* < *μ_c_*. The relaxation time *τ* can be given by $\tau =\mu E_{f}e^{-1}\upsilon_{F}{}^{-2}$, where the Fermi velocity *υ_F_* is 10^6^ m/s, *μ* is the carrier mobility assumed as 1310 cm^2^V^−1^s^−1^ for the graphene strips and 26,250 cm^2^V^−1^s^−1^ for the cross-shaped graphene, and the corresponding relaxation time is 0.105 ps and 2.1 ps with *E_f_* = 0.8 eV, respectively.

The complex relative permittivity of STO material is sensitive to the temperature, which can be expressed as follows [40,54]

(2) $$\varepsilon_{STO}=\varepsilon_{\infty }+\frac{f}{k_{0}^{2}-k^{2}-ik\gamma }$$

where *k* is the wave number of the incident wave, *ε*_∞_ = 9.6 is the high-frequency bulk permittivity, $k_{0}=\sqrt{31.2(T-42.5)}$ cm^−1^ is the wave number of the soft mode, *f* = 2.3 × 10^6^ cm^−2^ represents the oscillator strength, and *γ* = −3.3 + 0.094*T* cm^−1^ is the damping parameter of the soft mode. The external temperature *T* is set as 400 K in this work except when stated otherwise.

**Figure 2:**
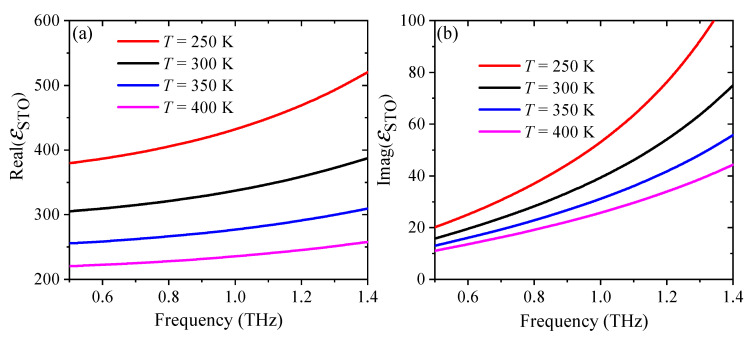
The simulated absorption spectra of (**a**) the individual upper metamaterial, and (**b**) the individual lower metamaterial.

In order to gain deep insight into the temperature-dependent property of STO, the real and imaginary parts of the permittivity as a function of frequency are calculated with various temperatures, as shown in Figure 2a,b, respectively. Both the real part and the imaginary part decreases with the increasing temperature. However, the value of the imaginary part is much smaller than that of the real part. The resonance frequency is mainly affected by the real part, and the imaginary part characterizes the losses. Therefore, the resonance frequency caused by the STO material can be significantly shifted by adjusting the environment temperature, while the intensity of the absorption changes little.

## 3. Results and Discussion

**Figure 3:**
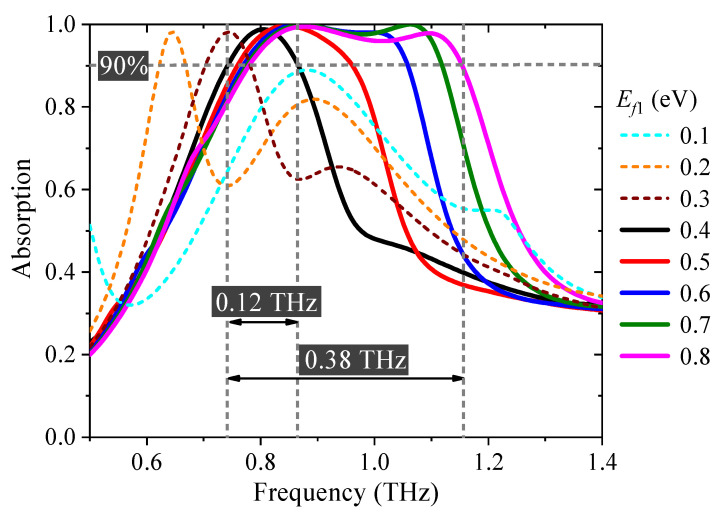
The absorption spectra with continuously tunable bandwidth. The Fermi energy level Ef1 changes from 0.1 to 0.8 eV.

The electromagnetic absorptance(*A*), i.e., the intensity of absorption, can be defined as *A* = 1 − *T* − *R*, in which transmittance *T* = |*S*_21_|^2^ and reflectance *R* = |*S*_11_|^2^ can be obtained from S-parameters in the simulation results calculated by COMSOL Multiphysics. When the Fermi energy level of graphene strips *E_f_*_2_ is set as 0.8 eV and that of the cross-shaped graphene *E_f_*_1_ is adjusted from 0.4 to 0.8 eV, the proposed absorber shows a tunable broadband absorption, as depicted in Figure 2. The bandwidth with the absorptance above 90% is 0.38 THz in the frequency range from 0.78 to 1.16 THz. As *E_f_*_1_ decreases to 0.4 eV, the bandwidth grows narrower and reaches the minimum 0.12 THz. The absorption broadband disappears and divides to become two absorption bands when *E_f_*_1_ continues to decrease, as depicted in Figure 3 by the dashed curves. Therefore, the bandwidth can be dynamically and continually tuned in the range of frequencies from 0.12 to 0.38 THz through controlling *E_f_*_1_.

**Figure 4:**
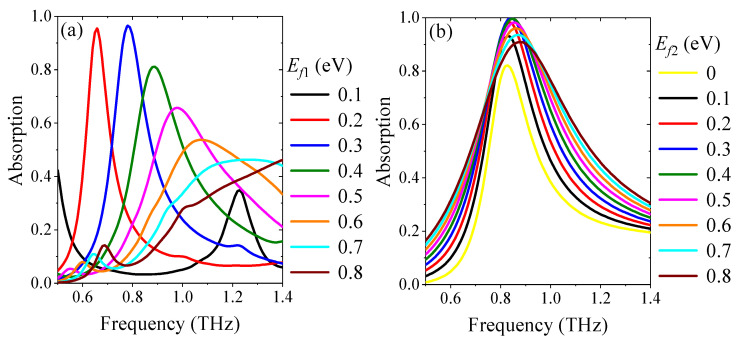
The simulated absorption spectra of (**a**) the individual upper metamaterial, and (**b**) the individual lower metamaterial.

To understand how the absorption broadbands are formed, the influences on absorption spectra of the individual upper and lower metamaterials are calculated, respectively. The absorption performance of the individual cross-shaped graphene with *E_f_*_1_ varying from 0.1 eV to 0.8 eV is shown in Figure 4a. When *E_f_*_1_ increases, the center frequency shows a monotonous blue-shift. The absorption enhancement starts to increase, then decreases, and achieves the maximum absorptance with *E_f_*_1_ = 0.3 eV. Thus, *E_f_*_1_ not only affects the location of the center frequency, but also the intensity of absorption. As shown in Figure 4b, the absorption intensity of the individual lower layer of STO combined with graphene strips is changed through adjusting *E_f_*_2_, while the variation of the center frequency is very small, which indicates that *E_f_*_2_ only influences the intensity of the absorption. The impedance-matching theory can be used as a physical explanation the effect of Fermi energy level on the absorption intensity of the individual upper and lower metamaterials. Consequently, due to the impedance matching between the free space and the proposed absorber, the upper and lower metamaterials combined effectively, which contributes to the stable high absorptance and the wide operating bandwidth, as depicted in Figure 2 by the solid curves.

**Figure 5:**
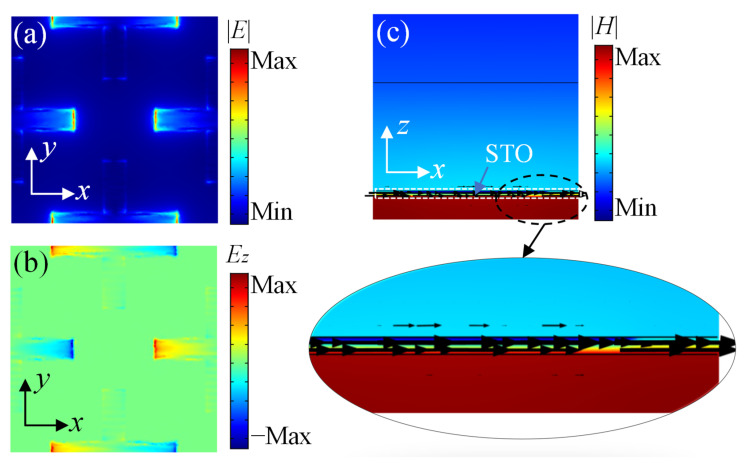
(**a**) Distribution of electric field amplitude |*E*| for the individual upper metamaterial. (**b**) Distribution of *z*-component of electric field *E_z_* for the individual upper metamaterial. (**c**) Distribution of magnetic field amplitude |*H*| and surface current (black arrows) for the individual lower metamaterial.

The distributions of the amplitude of electric field |*E*| and the *z*-component of electric field *E_z_* for the individual upper metamaterials are depicted in Figure 5a,b, respectively, at the resonance frequency of 0.78 THz with *E_f_*_1_ = 0.3 eV. The amplitude of |*E*| concentrates mainly around the ends and edges of the horizontal branches for the cross-shaped graphene. The distribution of *E_z_* shows that opposite charges accumulate at both ends of the horizontal branches, which indicates a typical electric dipole resonance. Figure 5c describes the distributions of magnetic field |*H*| as well as the surface current (black arrows) for the individual lower metamaterial at the resonance frequency 0.85 THz with *E_f_*_2_ set as 0.7 eV. The directions of the currents of the unit cell without the bottom gold block are marked by black arrows, while the magnetic field is concentrated below the STO layer, which demonstrates that there are induced currents on the gold layer. Then, a strong magnetic resonance is caused by these parallel surface currents with opposite direction. Therefore, the resonance absorptions of the individual upper and lower metamaterials can be attributed to the electric dipole resonance and the magnetic resonance, respectively.

**Figure 6:**
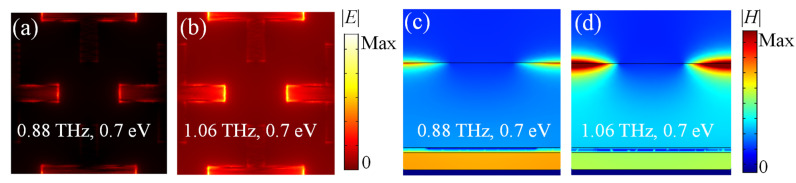
The distributions of electric field amplitude |*E*| for the cross-shaped graphene metamaterial (**a**) at 0.88 THz and (**b**) at 1.06 THz. The amplitude of magnetic field |*H*| of the central side view (**c**) at 0.88 THz and (**d**) at 1.06 THz.

The amplitude distributions of electric field and magnetic field at the resonance frequencies under normal TM polarized incidence are shown in Figure 6 to further investigate the merging of its upper and lower layer of metamaterials. When *E_f_*_1_ is 0.7 eV and *E_f_*_2_ is 0.8 eV, the first resonance frequency is 0.88 THz, and the second resonance frequency is 1.06 THz. The electric dipole resonance and the magnetic resonance coexist to originate the broadband absorption. For the cross-shaped graphene, the electric dipole resonance at the second resonance is much stronger than that at the first resonance, as shown in Figure 6a,b. Figure 6c,d shows that the magnetic resonance below the STO layer at 0.88 THz is more concentrated than that at 1.06 THz. Consequently, the upper metamaterial contributes more to the broadband absorbing performance at the second resonance, while the lower metamaterial contributes more to the broadband absorption at the first resonance.

**Figure 7:**
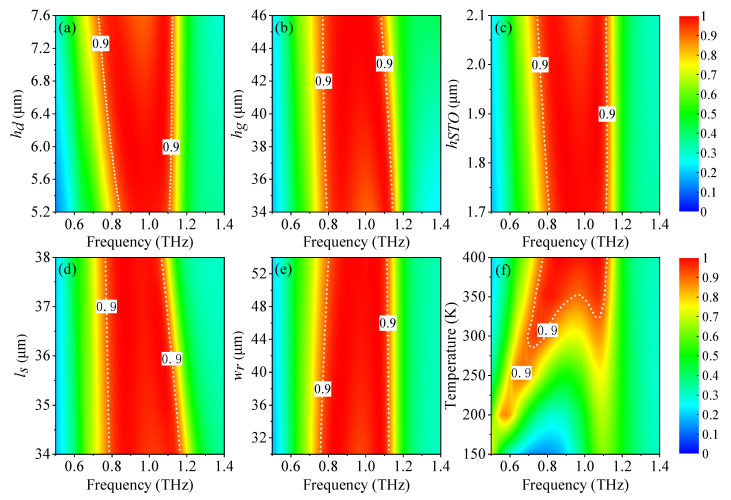
Absorption spectra varying with (**a**) the thickness *h_d_* of the spacer between the STO and the metallic ground, (**b**) the thickness *h_g_* of the spacer between the cross-shaped graphene metamaterial and the metallic ground, (**c**) the thickness *h_STO_* of STO, (**d**) the length of the graphene cross *l_s_*, (**e**) the width of the graphene strip *w_r_*, and (**f**) the temperature of STO. An absorptance of 90% is indicated by the white contour curves.

To comprehend the influence of the geometrical parameters on the broadband absorbing properties, the absorption spectra that vary with various parameters are simulated. The absorption spectra that vary with the thickness *h_d_* of the Topas layer between STO and the metallic ground is shown in Figure 7a. The location of the first resonance clearly shows a red-shift and the bandwidth grows wider gradually, which can be attributed to the fact that the increasing *h_d_* will affect the magnetic resonance below the STO layer. It is evident that the red shift of the second resonance frequency changes slightly and the bandwidth remains almost unchanged with the increase of the insulating spacer *h_g_* because the upper metamaterial contributes more to the broadband absorption at the second resonance, as shown in Figure 7b. When the thickness of the STO material layer *h_STO_* varies from 1.7 μm to 2.1 μm, the first absorption resonance has red shift, which results in the increment of bandwidth, as shown in Figure 7c. The absorption spectra of the branch length *l_s_* increasing from 34 μm to 38 μm is shown in Figure 7d. It is obvious that the second resonance has red shift with the increment of *l_s_*. That is because the resonance frequency varies inversely to the effective length of the patterned structure on the basis of the LC circuit model. Figure 7e shows that the bandwidth with an absorptance over 90% decreases slightly when the width of the graphene strip *w_r_* changes from 31 μm to 53 μm. The absorption spectra show quite a change when the temperature is lower than 375 K, which can be attributed to the influence of temperature on the conductivity of the STO material, as the impedance matching is destroyed with the drop in temperature, as shown in Figure 7f.

**Figure 8:**
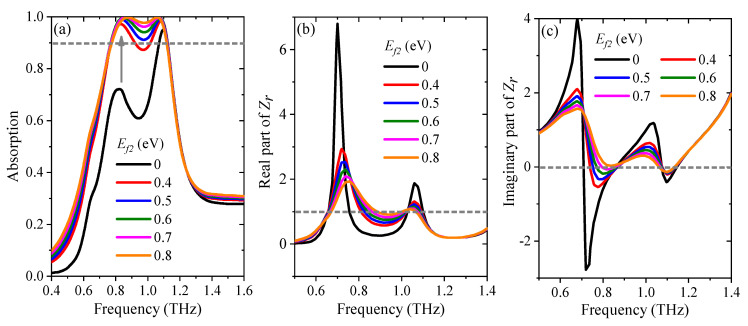
(**a**) The absorption spectra with various Fermi energy level of the graphene strips. (**b**) Real part and (**c**) imaginary part of the relative impedance *Z_r_* with various Fermi energy levels of the cross-shaped graphene.

Furthermore, Figure 8a shows that the first absorption peak decreases with the decline of *E_f_*_2_, while the location of the absorption peak remains unchanged. The Fermi energy level of the graphene strips only influences the flatness of the broadband absorption, and the impedance matching theory can be used to explained this phenomenon. The intensity of the absorption can reach the maximum when the relative impedance is 1. Figure 8b,c denotes the real and imaginary parts of the relative impedance *Z_r_* with *E_f_*_1_ changing in the range of 0.4–0.8 eV. When *E_f_*_2_ is set as 0.8 eV, the real part of *Z_r_* approaches 1, and the imaginary part of *Z_r_* approaches 0, respectively, in the frequency range from 0.78 to 1.16 THz, which indicates that the impedance matching of the proposed absorber and the free space, i.e., the maximum absorption intensity, can be achieved. The range of matching frequencies falls as the Fermi energy level decreases. The absorption bandwidth reaches the minimum as the Fermi energy level decreases to 0.4 eV.

**Figure 9:**
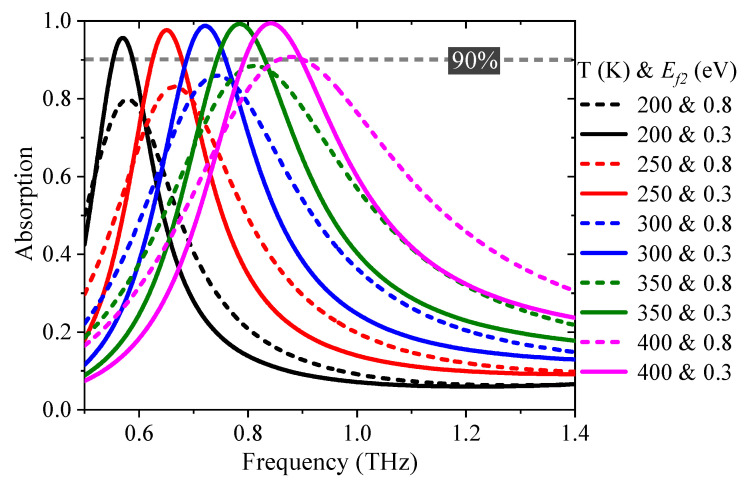
The absorption spectrum for a narrowband absorber with various temperatures. The dashed curves indicate the absorptance at *E_f_*_2_ = 0.8 eV, and the solid curves illustrate the absorptance at *E_f_*_2_ = 0.3 eV.

In addition, the broadband absorber can be transformed into a narrowband absorber when the *E_f_*_1_ is fixed as zero, as depicted in Figure 9. Attributed to the thermal control relative permittivity of STO, the center frequency is clearly blue-shifted from 0.58 THz to 0.88 THz when the external temperature rises from 200 to 400 K with *E_f_*_2_ = 0.8 eV, as shown by the dashed curves in Figure 9. The absorptance can be further improved through tuning *E_f_*_2_. The peak absorption intensity is over 96% at various temperatures with *E_f_*_2_ = 0.3 eV, as shown by the solid curves. Thus, a perfect narrowband absorber with a dynamically tunable center frequency can be achieved.

The effects of oblique incidence under both transverse electric (TE) and transverse magnetic (TM) waves on the absorption performance of the bifunctional absorber are finally investigated. The refraction angle *θ_m_* in layer m with the incident angle *θ_i_* can be expressed as *θ*_m_ = arcsin(*n*_1_sin(*θ_i_*)/*n*_m_) based on Snell’s law, where *n*_1_ and *n_m_* are the refractive index of the first and *m*-th layers. It is noteworthy that the refraction angle is a complex value when the *m*-th medium is a lossy material, which reflects that the refracted wave in the lossy material is a non-uniform plane wave [55]. Therefore, the overall reflection of the proposed absorber is then the superposition of the multiple reflections at different interfaces.

**Figure 10:**
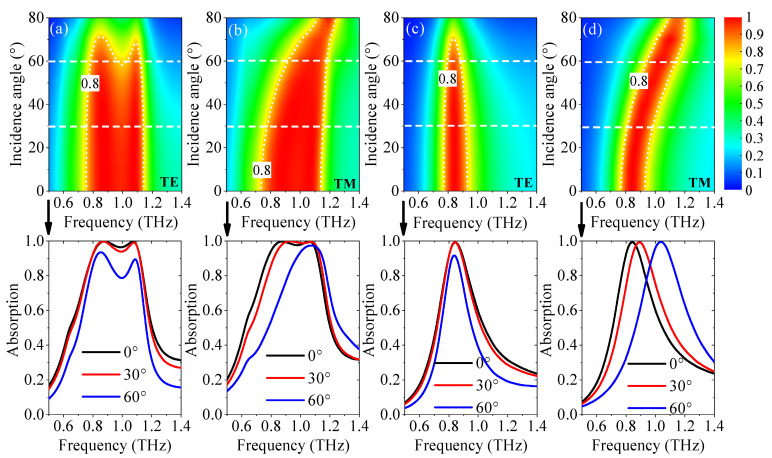
The spectrum of the broadband and narrowband absorption with various incidence angles for the TE wave (**a**,**c**) and the TM wave (**b**,**d**), respectively.

As shown in Figure 10a, when the bifunctional absorber acts as a broadband absorber, for the TE wave, more than 80% absorptance can be achieved for the incidence angle is up to 55°, and the center operating frequency remains unchanged. The closer the incidence angle to 90°, the smaller the absorptance. For TM polarization, the absorptance remains over 80% for the incidence angle up to 80°, as depicted in Figure 10b. When the incidence angle is over 40°, the center frequency shows blue shift clearly. The influence on absorption spectra is attributed to the change in zero-reflection condition under various incidence angles. For the narrowband absorption, the temperature is fixed at 400 K, and *E_f_*_2_ is set as 0.3 eV. As shown in Figure 10c,d, the absorptance remains above 80% with the incidence angle smaller than 80° for both TE and TM waves. With the increase in the incidence angle, the center frequency remains unchanged for the TE wave, while a clear blue shift is observed for TM polarization, which is consistent with the results in Figure 10a,b. Consequently, the bifunctional absorber is not sensitive to the polarization for wide oblique incidence angles.

## 4. Conclusions

In summary, a bifunctional absorber based on a graphene metamaterial and STO is designed with dynamically tunable and switchable properties. Compared with some other’s previous work, the bifunctional absorber presents excellent tunable ability. When *E_f_*_1_ varies from 0.4 eV to 0.8 eV, a broadband absorption is achieved, and its bandwidth varies from 0.12 THz to 0.38 THz with the intensity exceeding 90%. The results indicate that the broadband absorption benefits from the combination of graphene metamaterial and STO material. For the broadband absorption, the rate of change of the bandwidth is 216.6%, and that of the center frequency is 21%. The broadband absorber can be transformed into a narrowband absorber, when *E_f_*_1_ is set as 0. The center frequency of the narrowband absorption can be adjusted from 0.56 THz to 0.84 THz by controlling the temperature of STO. The peak intensity of the narrowband absorption is approximately 100% when *E_f_*_2_ is set as 0.3 eV, and the rate of change of the center frequency is 50%. The bifunctional absorber is not sensitive to polarization for large incidence angles. Hence, the proposed absorber may be suitable for many potential applications, for example sensing, optical cloaking, and some other switchable devices.

## Figures and Tables

**Table 1:** Comparison between our work and other terahertz absorbers with correlative functionality.

Ref.	Functionality	Dynamically Tunable Ability			
		BW (THz)	ROC of BW	*f_c_* (THz) with Absorptance > 90%	ROC of *f_c_*
[42]	Narrowband absorption	\	\	Tunable from 1.395 to 1.381	1%
[43]	Narrowband absorption	\	\	Tunable from 5.0 to 5.6	12%
[44]	Broadband absorption	Tunable 0.66 to 0.81 with BW > 80%	22.7%	Tunable from 1.325 to 1.555	17%
[45]	Broadband absorption	Fixed	\	Fixed	\
[46]	Broadband and narrowband absorption	Fixed	\	Broadband: fixed Narrowband: fixed	\
[47]	Broadband and narrowband absorption	Tunable from 0.725 (about) to 1.3 with BW > 90%	80%	Broadband: nearly fixed; Narrowband: tunable from 1.1 to 1.4 (about)	27% for narrowband
This work	Broadband and narrow-band absorption	Tunable from 0.12 to 0.38 with BW > 90%	216.6%	Broadband: tunable from 0.8 to 0.97; Narrowband: tunable from 0.56 to 0.84	21% for broadband; 50% for narrowband

## Data Availability

Data are available in the main text.
